# Concurrent Viral Transmission and Wildfire Smoke Events Following COVID-19 Pandemic School Closures in New York City: Associations of a Large Natural Experiment With Acute Care for Pediatric Asthma, 2018-2023

**DOI:** 10.1016/j.acepjo.2025.100273

**Published:** 2025-11-17

**Authors:** Sarah McCuskee, Rishi Kowalski, Cindy Mei, Alexis Zebrowski, Aman Patel, Lauren Zajac, Hashem Zikry, Ali Ayyash, Nicholas DeFelice

**Affiliations:** 1 Department of Emergency Medicine, Icahn School of Medicine at Mount Sinai, New York, New York, USA; 2 Arnhold Institute for Global Health at Mount Sinai, New York, New York, USA; 3 Institute for Exposomic Research, Icahn School of Medicine at Mount Sinai, New York, New York, USA; 4 Department of Environmental Medicine, Icahn School of Medicine at Mount Sinai, New York, New York, USA; 5 National Clinician Scholars Program, University of California, Los Angeles, California, USA; 6 Department of Emergency Medicine, University of California, Los Angeles, California, USA; 7 Department of Pulmonary, Sleep, and Allergy, Drexel University College of Medicine, Philadelphia, Pennsylvania, USA

**Keywords:** asthma, respiratory viruses, COVID-19 pandemic, environmental exposures, pediatrics, wildfire smoke

## Abstract

**Objectives:**

Pediatric asthma is multifactorial: social, environmental, and infectious exposures trigger exacerbations. School COVID-19 policies, including closures, mask mandates, and reopenings, provide a natural experiment to discover associations between the severity of pediatric asthma exacerbations, viral transmission, and exposure to wildfire smoke. We investigated the epidemiology of acute unscheduled care for asthma in children aged 5 to 17 years, including emergency department (ED) visits, hospital and intensive care unit (ICU) admissions, and viral testing.

**Methods:**

This observational study used a retrospective review of all electronic health records from New York City’s largest health system over 5 school years, from 2018 to 2023. Comparison groups included citywide surveillance data and all-cause visits. We investigated population-level exposures, including COVID-19 mitigation policies and wildfire smoke events.

**Results:**

In the postpandemic period, fewer children presented to the ED for asthma (*P* < .0001); however, more children required hospital or ICU admission (*P* < .001). Viral testing was employed more frequently and was more often positive in the postpandemic period (*P* < .0001). Rhinovirus was positive in a greater proportion of pediatric asthma visits than in all-cause adult or pediatric visits (*P* = .02). After mask mandates ended in early 2022, pediatric asthma visits peaked during the return-to-school fall period, slightly preceding the winter 2022 viral illness peak. Wildfire smoke events were not associated with pediatric asthma visits.

**Conclusion:**

Viral transmission and school policies were associated with pediatric asthma exacerbations during and after the COVID-19 pandemic. Increased acuity, despite lower volumes, may help guide health systems as they strive to increase readiness to care for pediatric populations in response to future pandemics or environmental exposures.

**The Bottom Line**

School closures and reopenings during the COVID-19 pandemic created a large natural experiment, causing sudden changes in the viral, environmental, and social triggers of pediatric asthma. After schools reopened, children in New York City were exposed to multiple wildfire smoke events. This observational review of acute care visits for pediatric asthma in New York City, from 2018 to 2023, included 2604 patients with 4311 visits. After schools reopened, the total number of visits decreased, but acuity increased, as more children required hospital or intensive care unit admission. Viral triggers were associated with visits, whereas wildfire smoke was not. These findings may increase the health system’s readiness to care for children.

## 1 Introduction

### 1.1 Background

Asthma is one of the most common chronic medical conditions in children, and its pathogenesis is multifactorial and complex. The importance of the built environment and social drivers of health is increasingly recognized,^1^, ^2^, ^3^, ^4^ and they are linked to the etiologies of asthma, including poor air quality, such as suspended particulate matter less than 2.5 μm, aeroallergens, and triggers in the home environment, such as mold, cockroaches, and other proinflammatory substances. All of these factors are socially driven.^3^ Additionally, exposure to respiratory viruses plays a substantial role in the pathogenesis and exacerbation of asthma symptoms, driving health care utilization and morbidity; virus transmission is itself socially driven.^5^ Together, these factors are likely responsible for a significant proportion of the known geographic, income, and racial/ethnic variability in pediatric asthma prevalence and morbidity.^4^^,^^6^ For these reasons, for example, children living in the lower-income neighborhood of East Harlem, New York City (NYC), have disproportionately high asthma morbidity compared with their geographically close neighbors.^7^

The COVID-19 pandemic markedly changed several of these social drivers of pediatric asthma.^5^ NYC had a well-defined citywide “New York on PAUSE” policy, including school closures, reopening with mandatory masking, and “demasking” (full opening without masking requirement). Previous literature has investigated pediatric asthma during the pandemic in parts of NYC,^5^ but the timing and impact of school closures, the “New York on PAUSE” policy, school reopenings, and demasking policies have not been investigated. During the COVID-19 pandemic, testing for respiratory viruses also became commonplace in emergency departments (EDs).^2^ This provides a natural experiment that allows the estimation of the impact of socially driven respiratory virus transmission on pediatric asthma morbidity in a large urban population.

Concomitantly, the NYC area experienced several unusually severe wildfire smoke events during the pandemic recovery period. Wildfire smoke has been associated with ill-health outcomes, particularly from respiratory causes. Despite ecological data supporting this link, studies examining the impact of wildfire smoke on pediatric asthma are heterogeneous and lack significant associations, as indicated in a recent meta-analysis.^8^ The effects of wildfire smoke on the pathophysiology and health system burden in pediatric asthma are not completely understood.

### 1.2 Importance

Understanding pediatric asthma epidemiology and health care utilization is critical for planning public health interventions. Undersampling of patients from groups exposed to multiple social drivers of disease is a known source of bias in pediatric asthma research.^9^ Selection bias may pose challenges to studies attempting to inform public health interventions and assess the overlapping impacts of multiple exposures. Therefore, we used observational data from the electronic health record (EHR) to mitigate selection bias and provide actionable estimates.^10^

### 1.3 Goals of This Investigation

We investigated the epidemiology, viral milieu, and environmental drivers of acute unscheduled care for pediatric asthma across 6 hospitals comprising the largest health system in NYC, in relation to the timing of policies intended to mitigate COVID-19 viral transmission over 5 school years, from 2018 to 2023.

## 2 Methods

### 2.1 Study Design and Setting

This was a retrospective observational study of ED visits and inpatient hospital admissions for asthma in children aged 5 to 17 years, using routinely collected EHR data from 5 hospitals across Manhattan, Queens, and Brooklyn, comprising the largest urban health system in NYC over the period 2018-2023.

The study was approved by the Institutional Review Board of the Icahn School of Medicine at Mount Sinai, STUDY-20-01285.

### 2.2 Selection of Participants

To form the study population, we included all acute unscheduled care, defined as ED, hospital, and intensive care unit (ICU) encounters, for pediatric asthma during the period from September 1, 2018, to June 27, 2023, which included all full school year periods between 2018 and 2023. Inclusion criteria were encounters for asthma, defined using the primary encounter diagnosis code by the International Classification of Diseases, 10th edition (ICD-10) as codes “J45.20,” “J45.21,” “J45.22,” “J45.30,” “J45.31,” “J45.32,” “J45.40,” “J45.41,” “J45.42,” “J45.50,” “J45.51,” “J45.52,” “J45.901,” “J45.902,” “J45.909,” “J45.990,” “J45.991,” “J45.998,” and pediatric age of the patient (children aged 5-17 years at the time of each encounter). Analyses were conducted both by patients and encounters, as some patients had repeat encounters. Hospital admissions and ICU stays occurring after transferring to the pediatric hospital from a different ED were linked using transfer data to the initial ED visit. Public figures and family members of health system employees were excluded, per institutional policy.

### 2.3 Interventions

This was an observational study; however, we leveraged the natural experiment of changing policies directed at children in NYC during the study period. NYC public schools were closed on March 15, 2020. Schools fully reopened for in-person instruction with a masking mandate in place on September 13, 2021. The masking mandate for schools was dropped effective March 8, 2022.

### 2.4 Measurements

Independent variables for the study were sex assigned at birth, health insurance, patient guardian-declared race/ethnicity, viral testing utilization, viral testing results, period of investigation (preschool closures, during school closures, postmasked reopening, postunmasking, and during wildfire smoke events), and environmental exposures (fine particulate air pollution [PM_2.5_] and pollen).

For each encounter, demographic (sex assigned at birth, insurance, patient guardian-declared race/ethnicity, and zip code of residence), utilization variables (length of stay, ED/hospital/ICU admission, and viral test administration, for which Current Procedural Terminology [CPT] codes are provided in Table S1), and clinical variables (viral test results) were collected. Two comparator populations were also extracted, using the same variables as the study population, within the 5 hospitals of the health system: all ED visits in pediatric (aged 5-17 years) and all-age populations over the same period.

A third comparison dataset was created to assess the representativeness of the detailed health system sample as well as infectious exposures. We extracted NYC Syndromic Surveillance data for the same periods, which indicates the total number of ED visits across NYC for either asthma or influenza-like illness (ILI+) in children aged 5 to 17 years. Data were provided by the NYC Department of Health and captured 100% of ED visits in the city.^11^ Visits were classified using text processing of chief complaints and ICD-10 discharge diagnosis codes. Asthma visits included ED visits among NYC resident children with ICD-10 primary encounter diagnosis code J45 or R06.2 or mention of “asthma, wheezing, or reactive airway.” ILI+ visits included mention of “flu, fever, and cough or sore throat.”

To assess environmental triggers of asthma, we extracted data on air quality, pollen, and weather. PM_2.5_ was extracted from sensor data available from the US Environmental Protection Agency Air Quality System Data Mart.^12^ We took the mean reading from sensors closest in proximity to the zip code tabulation areas represented in the patient dataset (a list of sensors is available in the supplementary material, Data source S1). Using these data, we identified 4 wildfire smoke event periods based on an Air Quality Index greater than 100 or “unhealthy for sensitive groups”^13^^,^^14^ (PM_2.5_ > 35.4 μg/m^3^/24 h) from 2019 to 2023 in NYC, and defined pre- and postevent 7-day periods. We confirmed the source of smoke during the identified periods by cross-referencing news reports and other published literature.^15^, ^16^, ^17^ We extracted measured pollen counts (grains/m^3^) from Fordham University’s midtown Manhattan air sampler stratified by source (tree, grass, and weed).^18^ Temperature and humidity were obtained by aggregating hourly values from the North American Land Data Assimilation System (NLDAS), averaged across five 0.125-degree grid cells that encompass Manhattan, where the majority of patients in the study population reside.^19^

### 2.5 Outcomes

The primary outcomes were asthma-related ED visits and inpatient hospital admissions in the study population across the 5 study periods. Secondary outcomes included ICU admission and length of stay.

The secondary outcome in the pediatric and adult health system comparison populations was all-cause ED visits. Secondary outcomes in the citywide syndromic surveillance comparison population were ED visits for asthma or ILI+.

### 2.6 Analysis

For school year-related analyses, the data were stratified into 5 periods using the NYC Public Schools Calendar: period 1 (September 5, 2018, to June 26, 2019), which serves as the prepandemic baseline; period 2 (September 5, 2019, to June 25, 2020); period 3 (September 16, 2020, to June 25, 2021); period 4 (September 13, 2021, to June 27, 2022); and period 5 (September 8, 2022, to June 27, 2023). Primary and secondary outcomes were examined for the study population across the 5 time periods and by relevant school closure, mask mandate, and reopening dates. We compared groups and trends over time (across school year periods) using chi-squared, Fisher’s exact, or Kruskal-Wallis tests, as appropriate. Analyses were conducted in R version 4.2 (R Foundation for Statistical Computing); air quality indices were calculated from standardized concentrations using the R package “con2aqi.”^20^

## 3 Results

### 3.1 Characteristics of Study Participants

This study included 2604 unique patients with 4311 visits for pediatric asthma within the health system across the 5-year study period. Demographics of the study population are presented in Table 1; 1451 (56%) patients were male. There was no change in pediatric asthma patient sex or race/ethnicity during the study period; 95% of children were identified by parents/guardians as having a race or ethnicity other than White. Comparison groups included all-age (N = 394,020) and pediatric (aged 5-17 years, N = 20,931) visits across the health system over the study period, as well as comprehensive syndromic surveillance data for health facilities in NYC.

**Table 1:** Demographics of the study population: health system patients aged 5 to 17 years with an acute care visit for asthma, school years from 2018 to 2023.

School year (dates)	Period 1	Period 2	Period 3	Period 4	Period 5	*P*
	9/5/18-6/26/19	9/5/19-6/25/20	9/16/20-6/25/21	9/13/21-6/27/22	9/8/22-6/27/23	
	N (%)					
Total unique patients	1044 (100)	682 (100)	280 (100)	587 (100)	712 (100)	<.0001
Sex						
Male	595 (57)	385 (56)	155 (55)	331 (56)	398 (56)	.987
Female	449 (43)	297 (44)	125 (45)	256 (44)	314 (44)	
Visits per child, mean (SD)						
Male	1.34 (1.05)	1.27 (0.85)	1.21 (0.61)	1.31 (0.78)	1.36 (1.02)	.907
Female	1.33 (0.92)	1.27 (0.87)	1.19 (0.73)	1.27 (0.87)	1.32 (0.91)	
Race/ethnicity						
Black/African American	429 (41)	293 (43)	142 (51)	262 (45)	332 (47)	.346
White	29 (2.8)	30 (4.4)	7 (2.5)	14 (2.4)	22 (3.1)	
Asian American/Pacific Islander	21 (2.0)	9 (1.3)	3 (1.1)	11 (1.9)	9 (1.3)	
Other	135 (13)	87 (13)	33 (12)	78 (13)	86 (12)	
Hispanic/Latino	418 (40)	253 (37)	94 (34)	218 (37)	254 (36)	
Unknown	12 (1.1)	10 (1.5)	1 (0.36)	4 (0.68)	9 (1.3)	
Insurance coverage						
Medicare	0 (0.0)	0 (0.0)	0 (0.0)	1 (0.17)	1 (0.14)	<.0001
Medicaid	483 (46)	309 (45)	138 (49)	353 (60)	461 (65)	
Commercial	120 (12)	81 (12)	31 (11)	87 (15)	113 (16)	
Self-pay	2 (0.2)	2 (0.29)	1 (0.36)	0 (0.0)	0 (0.0)	
Other	439 (42)	290 (43)	110 (39)	146 (25)	137 (19)	

Changes in insurance over the study period may reflect changing patient registration practices. Statistical tests are chi-squared, except where expected values are <5, in which case Fisher’s exact test was used. For changes in mean visits per child by sex over time, statistical testing was performed using analysis of variance.

### 3.2 Main Results

#### 3.2.1 Asthma-related health care utilization in the study population

As presented in Table 2, from the beginning to the end of the 5-year study period, pediatric ED visits for asthma declined in the health system study population (total of 1393 visits in period 1 vs 955 in period 5; *P* for all years < .0001). After a precipitous drop during the 2019-2020 school year, visits increased but did not rebound to their prepandemic 2018-2019 (period 1) baseline. However, asthma-related hospital admissions and ICU stays increased during and postpandemic, both in absolute terms and as a proportion of ED visits (hospital admissions: 62 in period 1 vs 87 in period 5; ICU admissions: 16 in period 1 vs 25 in period 5; *P* for all years < .001).

**Table 2:** Acute care visits for asthma in children aged 5 to 17 years in a large New York City health system during the school years from 2018 to 2023, including sex-stratified acuity and viral testing.

School year (dates)	Period 1	Period 2	Period 3	Period 4	Period 5	***P***
	9/5/18-6/26/19	9/5/19-6/25/20	9/16/20-6/25/21	9/13/21-6/27/22	9/8/22-6/27/23	
	N (%)					
Total visits	1393 (100)	867 (100)	336 (100)	760 (100)	955 (100)	<.0001
ED-only (% total)	1331 (96)	828 (96)	307 (91)	713 (94)	868 (91)	<.0001
Admitted to hospital (% total)	62 (4.5)	39 (4.5)	29 (8.6)	47 (6.2)	87 (9.1)	
Admitted to ICU (% total)	16 (1.2)	8 (0.92)	11 (3.3)	26 (3.4)	25 (2.6)	
Sex-stratified acuity						
ED-only visits						
Male (% ED)	765 (57)	472 (57)	177 (58)	410 (57)	495 (57)	.999
Female (% ED)	566 (43)	356 (43)	130 (42)	303 (43)	373 (43)	
Admitted to the hospital						
Male (% hospital)	21 (46)	17 (55)	9 (50)	10 (48)	32 (52)	
Female (% hospital)	25 (54)	14 (45)	9 (50)	11 (52)	30 (48)	
ICU admission						.054
Male (% ICU)	8 (50)	4 (50)	10 (91)	13 (50)	9 (36)	
Female (% ICU)	8 (50)	4 (50)	1 (9)	13 (50)	16 (64)	
Viral testing						<.0001
Any viral test administered (% total)	134 (9.6)	126 (15)	191 (57)	523 (69)	680 (71)	
Any positive viral test (% total)	17 (1.2)	28 (3.2)	9 (2.7)	110 (14)	141 (15)	<.0001

Statistical tests are chi-squared, except where expected values are <5, in which case Fisher’s exact test was used.

ED, emergency department; ICU, intensive care unit.

We investigated sex differences in asthma presentations, as noted in Table 2. Overall, male children accounted for a greater proportion of ED visits for asthma, with no significant change in proportion over time. Hospital admissions were not significantly different between males and females over the study period. However, ICU admissions were higher in female children, particularly in the final year of the study period, 2022-2023, when 16/25 children (64%) were female (*P* = .054).

#### 3.2.2 Health system ICU admissions

ICU admissions for asthma increased in the postpandemic period. Table 3 compares patients with asthma diagnoses admitted to the ICU with all ICU admissions in patients aged 5 to 17 years. Overall, there was an increase in the proportion of children admitted to the ICU for asthma (eg, 9.4% in period 1 vs 21.9% in period 5; *P* for change over time = .004). In the later study periods, most children admitted to the ICU were tested for at least 1 virus; the proportion of those testing positive increased over the study period, peaking in the 2021-2022 school year, with 84.6% of patients admitted to the ICU for asthma testing positive for a virus (*P* < .0001). Among ICU patients who received viral testing, there were no differences in sex, race/ethnicity, or insurance over the study period (Table S2). Overall, as shown in Table 4, the mean ICU length of stay was shorter for patients with asthma across all 5 years of the study (138 [SD, 301.4] hours vs 42.7 [SD, 26.2] hours; *P* < .0001). ICU patients who tested positive for a virus had longer lengths of stay than those who tested negative, both for nonasthma and for asthma ICU patients.

**Table 3:** Characteristics of intensive care unit admissions for all causes and asthma in children aged 5 to 17 years in a large New York City health system for the school years from 2018 to 2023: characteristics by school year.

School year (dates)	Period 1	Period 2	Period 3	Period 4	Period 5	*P*
	9/5/18-6/26/19	9/5/19-6/25/20	9/16/20-6/25/21	9/13/21-6/27/22	9/8/22-6/27/23	
	N (%)					
Admission totals						
ICU admissions: total all-cause, N	170	98	100	127	114	<.0001
ICU admissions: asthma (% total all-cause)	16 (9.4)	8 (8.2)	11 (11.0)	26 (20.5)	25 (21.9)	.004
ICU admissions tested (% total all-cause)	60 (35.2)	46 (47.0)	92 (92.0)	123 (96.9)	103 (90.4)	<.0001
ICU patients tested positive for any virus (% total all-cause)	14 (8.2)	9 (9.2)	12 (12.0)	56 (44.1)	34 (29.8)	<.0001
Asthma ICU admissions tested (% total asthma ICU admissions)	9 (56.3)	4 (50)	11 (100)	26 (100)	23 (92)	<.0001
Asthma ICU admissions tested positive for any virus, N (% total asthma, % asthma tested)	1 (6.3, 11.1)	1 (12.5, 25.0)	3 (27.2, 27.2)	22 (84.6, 84.6)	12 (48.0, 52.5)	<.0001
Admissions coinfected with/multiple viruses (% total all-cause)	2 (1.2)	0 (0)	0 (0)	6 (4.7)	2 (1.8)	-

Statistical tests are chi-squared, except where expected values are <5, in which case they are Fisher’s exact test.

ICU, intensive care unit.

**Table 4:** Intensive care unit length of stay by viral infection and asthma diagnosis among those who had viral testing across all school years from 2018 to 2023.

Admission type and group	LOS (h), mean (SD)	P
Non-asthma ICU admissions		
All	138 (301)	
No positive viral test	90.8 (221)	.029
Any positive viral test	220 (1050)	
Asthma ICU admissions		
All	42.7 (26.2)	
No positive viral test	30.0 (23.9)	.080
Any positive viral test	43.2 (31.6)	

Statistical tests for hospital LOS are the Kruskal-Wallis test.

ICU, intensive care unit; LOS, length of stay.

#### 3.2.3 Citywide ED visit comparison

**Figure 1:**
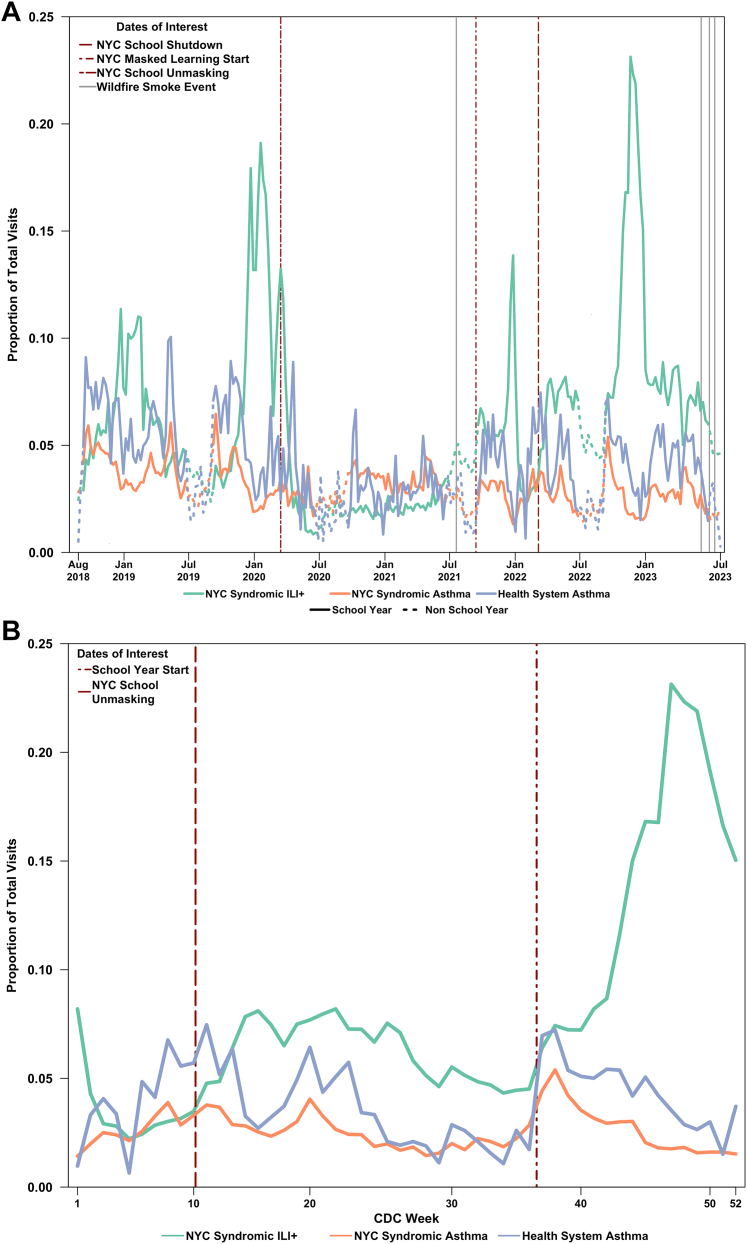
Proportion of total emergency department visits for asthma in health system data (blue) and asthma (orange) or influenza-like illness (ILI+, green) in New York City (NYC)-wide syndromic data in children aged 5 to 17 years for (A) 2018-2023, with solid lines during the school year and dashed during summers, and (B) 2022, showing an increasing incidence in asthma, followed by ILI+, at the start of the school year, after unmasking. CDC, Centers for Disease Control and Prevention.

Compared with the NYC-level pediatric population, the ED visit volume in the study population was similar to the NYC data for asthma syndrome visits, but differed from the NYC data for ILI+. The calendar year 2022 provides a natural experiment on the impact of masking in schools. NYC public schools required their students to wear a mask until March 8, 2022, when the policy shifted to masks being optional. In September 2022, schools reopened for the first school year without restrictions related to COVID-19. We plotted ED visits from the NYC-wide syndromic surveillance data for asthma and ILI+, as well as health system ED visits for asthma in the 5- to 17-year age group, in Figure 1. Seasonal variation was expected, as shown in Figure 1A, which demonstrates lower visits for both asthma and ILI+ during nonschool year periods (dashed trend lines). The fall 2022 peak in asthma was slightly preceded by the winter 2022 peak in ILI+, which occurred almost immediately during the return-to-school period (Fig 1B).

#### 3.2.4 Health system viral testing comparison

**Figure 2:**
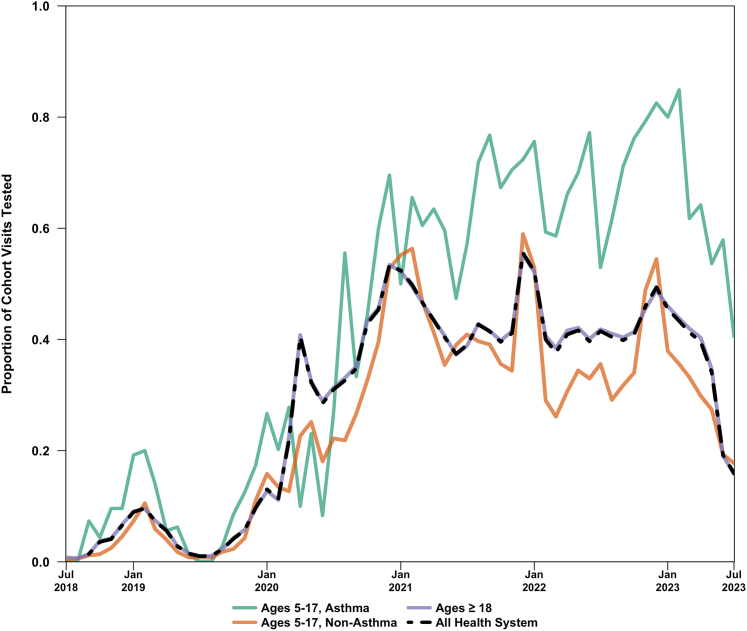
Monthly proportion of urban health system emergency department patients administered any viral test in pediatric (aged 5-17 years), pediatric asthma, and adult populations, from July 2018 to July 2023.

Figure 2 shows the proportion of patients who were administered any viral test, stratified by group (study population of pediatric asthma and nonasthma pediatric visits; comparator populations: visits of patients aged ≥ 18 years and across the entire health system). In the study population, viral testing was employed post-2020 with greater frequency (eg, in period 1, 9.6% were tested vs period 5, when 71% were tested; *P* for all periods < .0001) and was more likely to be positive (eg, 1.2% of all ED visits were positive for a virus in period 1 vs period 5, when 15% were positive; *P* for all periods < .0001). The increase in viral testing post-2020 was particularly prominent in children aged 5 to 17 years with asthma, whereas all pediatric visits more closely tracked testing in the population aged ≥ 18 years within the health care system. Testing administered during the study period, by type, is presented in Table S3.

**Figure 3:**
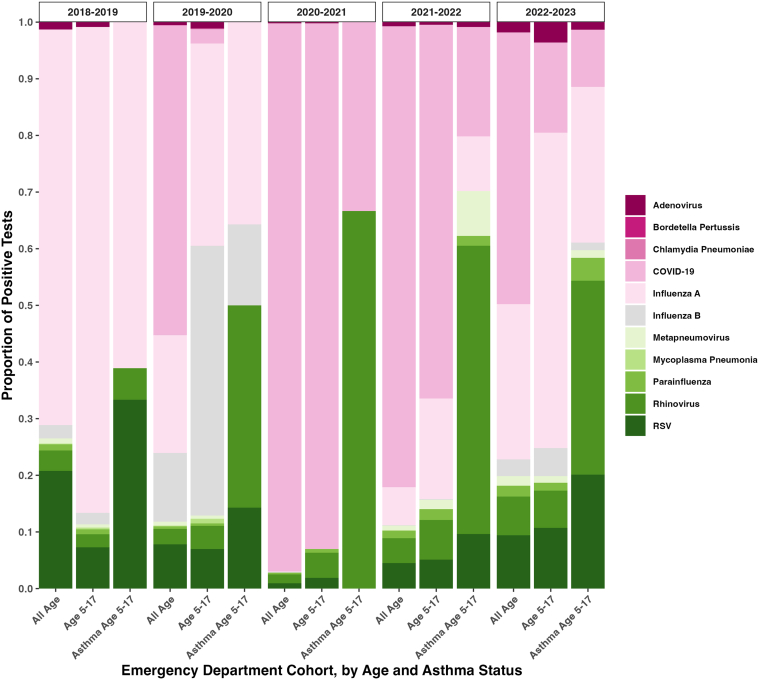
Viral testing results by emergency department patient cohort: pediatric (aged 5-17 years), pediatric asthma, and adult populations, in a large New York City health system, by school years from 2018 to 2023. RSV, respiratory syncytial virus.

Viral triggers for asthma differed from viral triggers for all-cause ED visits and hospitalizations in both the pediatric and all-age health system comparator populations (Fig 3). COVID-19 and influenza A and B accounted for large proportions of both pediatric and all-age ED visits and hospitalizations in all comparator school year periods. However, unique to pediatric asthma visits, rhinovirus appeared to be an important contributor every year after 2019, comprising 34% to 67% of viruses identified in pediatric asthma patients each year, although never accounting for more than 10% of all-cause adult and pediatric visits (*P* for difference between asthma and all-cause visits = .02).

#### 3.2.5 Environmental exposures

During the study period in NYC, wildfire smoke events occurred near or after the end of the school year on July 20, 2021, June 6 to 8, 2023, June 29 to 30, 2023, and July 14, 2023. In pediatric asthma patients presenting to the health system, there was no statistically significant difference in ED visit rates between the 7 days pre-event, the event period, and the 7 days postevent (Table 5). Grass pollen was lower in the postevent period (mean, 1.91 [SD, 2.56] grains/m^3^) vs the pre-event period (mean, 3.65 [SD, 4.5] grains/m^3^; *P* for all periods = .024). Sex differences were observed in postwildfire asthma presentations (70.7% male vs pre-event 42.6% male; *P* = .006). No race/ethnicity differences were observed. Temporal trends for environmental exposures are presented in Figure S1.

**Table 5:** Selected exposures and demographic variables for pediatric (aged 5-17 years) asthma visits pre-, during, and post-wildfire-related acute air quality events (PM_2.5_ > 35.4 μg/m^3^/24 h) in New York City.

	Before exposure	Exposure	After exposure	*P*
Airborne exposures	Mean (SD)			
Daily PM2.5 Air Quality Index	42.9 (12.9)	144.7 (54.5)	58.5 (21.1)	<.0001
Daily PM2.5 (μg/m^3^)	10.5 (3.6)	71.1 (52.5)	16.8 (8.5)	<.0001
Daily tree pollen count (grains/m^3^)	27.1 (41.9)	64.7 (78.6)	8.07 (6.11)	.524
Daily grass pollen count (grains/m^3^)	3.65 (4.5)	6.79 (5.7)	1.91 (2.56)	.024
Daily weed pollen count (grains/m^3^)	2.31 (3.27)	1.28 (2.07)	1.27 (2.08)	.564
NYC Syndromic Surveillance	Mean (SD)			
Daily NYC all-age visits for asthma	158 (20.6)	203 (71.1)	162 (31.0)	.203
Daily NYC all-age visits for ILI+^a^	208 (52.7)	212 (33.2)	204 (37.6)	.798
Health system nonasthma visits, aged 5-17 y	Mean (SD)			
Daily ED visits	14.0 (6.12)	11.6 (5.83)	12.1 (6.19)	.471
Daily ED visits with a positive viral test	1.29 (1.18)	0.71 (1.11)	1.46 (1.20)	.257
Health system asthma visits, aged 5-17 y	Mean (SD)			
Daily visits (ED, hospital, ICU)	1.82 (1.93)	2.29 (2.14)	1.50 (1.37)	.645
ED visits	1.68 (1.74)	2.29 (2.14)	1.39 (1.23)	.592
Daily visits (ED, hospital, ICU), positive viral test	0.107 (0.32)	0.14 (0.38)	0.071 (0.26)	.816
Sex	N (%)			
Male	20 (42.6)	5 (31.2)	29 (70.7)	.006
Female	27 (57.4)	11 (68.8)	12 (29.3)	
Race/ethnicity	N (%)			
Black	23 (48.9)	9 (56.2)	20 (48.8)	.587
White	1 (2.1)	1 (6.2)	0 (0)	
Asian American and Pacific Islander	0 (0)	1 (6.2)	2 (4.9)	
Other	4 (8.5)	1 (6.2)	4 (9.8)	
Hispanic/Latino	19 (40.4)	4 (25.0)	14 (34.1)	
Unknown	0 (0)	0 (0)	1 (2.4)	

Statistical tests are Kruskal-Wallis tests, except for the Race/Ethnicity comparison, which is Fisher’s exact test.

ED, emergency department; ICU, intensive care unit; ILI+, influenza-like illness; NYC, New York City; PM_2.5_, particulate matter <2.5 micrometers diameter.

a ILI+: viral syndromes, as described in the text.

## 4 Limitations

This observational study has several limitations. The associations presented may not be causal due to the observational design. Although using routine health system data allowed us to minimize inclusion biases, differences in medical coding of asthma visits driven by a viral infection may contribute to misclassification bias in patients with asthma if a visit for asthma exacerbation had a principal diagnosis code related to the viral etiology. However, NYC-wide syndromic surveillance ILI+ data, which uses free-text search in addition to ICD-10 codes, showed a very similar pattern to our cohort, suggesting that the risk of misclassification may be minimal. We also did not include asthma severity history due to incomplete data on chronic or outpatient disease severity within the EHR records for acute unscheduled care encounters. We did not observe racial/ethnic changes within our study population over time during our study period. This may reflect the largely minoritized study population. This proportion varies substantially across geographic areas in the literature,^21^ but appears representative based on other literature from NYC.^4^ Differences in sex should be further explored,^8^ as should geographic and systematic disparities, which have been repeatedly demonstrated in existing literature. Overall, although our results are similar to those observed in other urban settings in the United States^5^^,^^22^^,^^23^ and other countries,^24^^,^^25^ they may not be generalizable to all settings across the United States due to differences in viral and environmental exposures between urban and rural settings.

## 5 Discussion

This study underscores the importance of respiratory viral transmission as one of multiple dynamic seasonal risks in pediatric asthma, using the natural experiment of the COVID-19 pandemic and the policies intended to mitigate it. Despite the lower total ED volume of asthma in the pediatric population, those children who did present were significantly more likely to require hospital admission and ICU care. Multiple factors may contribute to this, including changes in care-seeking behavior during and postpandemic, changes in telemedicine or home management strategies undertaken by health care professionals, such as nebulizer or steroid prescriptions, which enable longer self-management of exacerbations, and differential exposures.^26^^,^^27^ COVID-19 itself was not associated with a majority of pediatric asthma presentations in any school year across the study period. However, positivity for any virus in ICU patients was associated with longer length of stay in both asthma and nonasthma presentations. These trends have implications for health system resource planning, suggesting that strengthening ICU surge capacity may improve care for this biologically susceptible patient population.

Our finding that male children were more likely to have ED presentations for asthma is consistent with most existing literature, but the trend toward increased ICU admissions in female children has not frequently been demonstrated.^28^^,^^29^ One large study found that a history of pneumonia was more common in girls with asthma than in boys with asthma^29^; given the importance of infectious (viral) triggers in our cohort, further research could investigate sex differences in relation to infectious asthma exacerbation triggers.

Clinical practice in the application of viral testing, including multiplex viral testing, became more liberal during the study period,^30^ which is likely to have influenced the distribution of viruses detected from 2018 to 2023. As shown in Table 2 and Figure 2, an increase in test administration (from 15% to 57%) occurred between period 2 and period 3, preceding the increase in test positivity (from 2.7% in period 3 to 14%-15% in periods 4 and 5). Given this lag, it is unlikely that the observed increase in viral positivity in the study population was solely due to increased testing. However, pediatric asthma patients had a distinct viral milieu within each school year compared with the general pediatric and all-age health system populations. As expected, the most frequently identified viruses in the all-age and pediatric ED visits were COVID-19 and influenza A and B. However, viral triggers in the pediatric asthma population were distinct, particularly the importance of rhinovirus. This has been previously documented in other literature^28^^,^^29^; however, its persistence as an important driver during both COVID-19 surges and the winter 2022 COVID-19/influenza/respiratory syncytial virus (RSV) surge is notable. The relative lack of rhinovirus detection in 2018-2019 may reflect the more limited testing strategy employed prior to the COVID-19 pandemic (Fig 2, Table 2, and Table S3). The increased use of viral testing in the pediatric asthma population, compared with adult and nonasthma populations, as shown in Figure 2, may reflect clinicians’ understanding of the epidemiologic literature to which our study adds, highlighting the importance of viral transmission as a driver of pediatric asthma exacerbations. Longitudinal research, which continues to examine the viral milieu in the pediatric asthma population, may help further clarify any longer-term impacts of the COVID-19 pandemic-related immunity debt and assess changes in viral and environmental drivers of pediatric asthma.^31^^,^^32^

Policies such as school closure/reopening and masking/demasking, intended to mitigate COVID-19 spread, appeared to have significant associations with pediatric asthma presentations, possibly driven by respiratory virus transmission. After school mask mandates were dropped in spring 2022, there were few pediatric asthma presentations during the summer of 2022. However, the return to school in fall 2022 was accompanied by surges in asthma, followed by ILI+ (Fig 1B). The timing of these 2 peaks may reflect the increased vulnerability of the pediatric asthma population to exposures, including viral transmission in schools or unmeasured competing risks, although exposures, including particulate air pollution, PM_2.5_, ozone, nitrogen dioxide, temperature, humidity, and pollen count, did not appear to be substantially different during this period (Fig S1). Nonpharmaceutical interventions, such as masking, may mitigate health care utilization for sensitive groups.

Pediatric asthma has many potential triggers, which vary seasonally, including the viral milieu and indoor allergens,^33^ as well as responses to policies and unexpected events, such as pollution sources like long-distance wildfire smoke, traffic emissions, residential combustion, and industrial processes. Exposure to indoor allergens was not measured in this study, but multiple studies, including ours, have demonstrated decreased acute care needs for pediatric asthma during periods when children were spending significant time indoors in their homes (pandemic remote learning).^5^^,^^22^^,^^23^ Compared with strong associations with viral transmission and positivity, this suggests that indoor home allergens may be less important as acute drivers of pediatric asthma exacerbation. Importantly, this does not imply that asthma incidence and pathogenesis are not linked to indoor aeroallergens, which are prevalent in NYC.^3^^,^^34^ In contrast to some prior studies,^16^ but in keeping with others,^35^ our research did not find significant differences in pediatric asthma or ILI+ presentations across the time periods pre-, during, and postwildfire smoke events. This may reflect the sensitivity of pediatric asthma to different choices of reference periods, given the multiple competing exposures present, or changes in behavior during highly publicized wildfire smoke events.^15^ In particular, seasonal effects may influence the relationship between wildfire smoke and pediatric asthma presentations. Because all events occurred during the same season in our study period, seasonal effects and competing exposures may mask an underlying relationship. Postpandemic modifications to school indoor air environments, for example, the provision of air filters in classrooms after 2021,^36^ may also influence children’s susceptibility to poor air quality, which has been demonstrated to worsen test scores.^37^ Other literature has demonstrated that wildfires are associated with increases in viral transmission in all-age populations,^38^ a mixture of exposures that should be investigated in pediatric asthma.

In summary, this study underscores the importance of policy decisions in influencing the complex mixture of social and environmental exposures that drive pediatric asthma in a diverse urban population. Leveraging the natural experiment of school closures, reopenings, and mask mandates, alongside changes in clinical practice that increased testing, we demonstrate the importance of viral transmission in pediatric asthma epidemiology and the increase in severe presentations of pediatric asthma in the post–COVID-19 period. Although widely observed, the postpandemic increase in pediatric asthma severity is incompletely understood, with implications for health system readiness and resource planning. Future work on pediatric asthma should consider its severity in the context of the mixtures of dynamic seasonal and static environmental risks.

## Author Contributions

ND conceived the study and secured research funding. SM, ND, and RK designed the study and conducted analyses. CM, AP, AZ, and RK extracted and cleaned the data. SM drafted the manuscript, and all authors contributed substantially to its revision. SM takes responsibility for the paper as a whole.

## Funding and Support

This work is supported by a K12 Pediatric and Reproductive Environmental Health Scholars award from the 10.13039/100000066National Institute of Environmental Health Sciences, K12-ES033594. This work is also part of the NIEHS-funded Center on Health and Environment Across the LifeSpan (HEALS), P30ES02351, and the NICHD K25 HD109509.

## Conflict of Interest

All authors have affirmed they have no conflicts of interest to declare.
