# Supplementary material for: Concurrent Viral Transmission and Wildfire Smoke Events Following COVID-19 Pandemic School Closures in New York City: Associations of a Large Natural Experiment With Acute Care for Pediatric Asthma, 2018-2023
Source: J Am Coll Emerg Physicians Open. 2025 Nov 17;7(1):100273. doi: 10.1016/j.acepjo.2025.100273 (PMC12666338; doi:10.1016/j.acepjo.2025.100273)
Supplement: Supplementary Tables 1-3 and Supplementary Figure 1 [file mmc1.docx]

**Data source S1:**

Fine particulate air pollution (PM2.5) were extracted from sensor data available on the US Environmental Protection Agency Air Quality System Data Mart. Sensors included were:

0080, 0135, 0110, 0112, 0079, 0128, 0122, 0134, and 0118.

*Table S1: Viral tests and associated Current Procedural Terminology (CPT®) codes and local health system procedure codes (when CPT not applicable) included in the study.*

| Panel/Lab name | Component name | CPT Code | Local Code |
| --- | --- | --- | --- |
| ADENOVIRUS QUANT PCR | ADENOVIRUS QUANT PCR | 87799 | - |
| BORDETELLA PCR | BORDETELLA PERTUSSIS BY PCR | 87798 | - |
| INFLUENZA A/B/RSV BY PCR | INFLUENZA A PCR | 87798 | - |
| INFLUENZA A/B/RSV BY PCR | RSV PCR | 87798 | - |
| INFLUENZA A/B/RSV BY PCR | INFLUENZA B PCR | 87798 | - |
| MANUAL SARS COV-2 PLUS FLU A & B PCR PANEL (POCT) LIAT | INFLUENZA A | 87636 | - |
| MANUAL SARS COV-2 PLUS FLU A & B PCR PANEL (POCT) LIAT | INFLUENZA B | 87636 | - |
| MYCOPLASMA PNEU. DNA DETECT | MYCOPLASMA PNEUMONIAE PCR | 87581 | - |
| PARAINFLUENZA PCR | PARAINFLUENZA 1 PCR | 87279 | - |
| PARAINFLUENZA PCR | PARAINFLUENZA 2 PCR | 87279 | - |
| PARAINFLUENZA PCR | PARAINFLUENZA 3 PCR | 87279 | - |
| RAPID FLU TEST (POCT) | RAPID INFLUENZA A | 87804 | - |
| RAPID FLU TEST (POCT) | SARS COV-2 PCR (POCT) LIAT | 87804 | - |
| RAPID FLU TEST (POCT) | RAPID INFLUENZA B | 87804 | - |
| RAPID FLU TEST (POCT) | INFLUENZA B | 87804 | - |
| RAPID FLU TEST (POCT) | INFLUENZA A | 87804 | - |
| SEVERE ACUTE RESPI SYNDROME CORONAVIRUS 2 (SARS-COV-2)(POCT) | INFLUENZA B | 87635 | - |
| SEVERE ACUTE RESPI SYNDROME CORONAVIRUS 2 (SARS-COV-2)(POCT) | INFLUENZA A | 87635 | - |
| SEVERE ACUTE RESPI SYNDROME CORONAVIRUS 2 (SARS-COV-2)(POCT) | SARS COV 2 PCR | 87636 | - |
| SEVERE ACUTE RESPI SYNDROME CORONAVIRUS 2 (SARS-COV-2)(POCT) | SARS COV 2 PCR | 87635 | - |
| SEVERE ACUTE RESPI SYNDROME CORONAVIRUS 2 (SARS-COV-2)(POCT) | INFLUENZA B | 87636 | - |
| SEVERE ACUTE RESPI SYNDROME CORONAVIRUS 2 (SARS-COV-2)(POCT) | INFLUENZA A | 87636 | - |
| RESPIRATORY PATHOGENS PANEL BY PCR | ADENOVIRUS PCR | - | 105489 |
| RESPIRATORY PATHOGENS PANEL BY PCR | BORDETELLA PERTUSSIS PCR | - | 105489 |
| RESPIRATORY PATHOGENS PANEL BY PCR | CHLAMYDOPHILA PNEUMONIAE PCR | - | 105489 |
| RESPIRATORY PATHOGENS PANEL BY PCR | INFLUENZA A PCR | - | 105489 |
| RESPIRATORY PATHOGENS PANEL BY PCR | INFLUENZA B PCR | - | 105489 |
| RESPIRATORY PATHOGENS PANEL BY PCR | METAPNEUMOVIRUS PCR | - | 105489 |
| RESPIRATORY PATHOGENS PANEL BY PCR | MYCOPLASMA PNEUMONIAE PCR | - | 105489 |
| RESPIRATORY PATHOGENS PANEL BY PCR | PARAINFLUENZA 1 PCR | - | 105489 |
| RESPIRATORY PATHOGENS PANEL BY PCR | PARAINFLUENZA 2 PCR | - | 105489 |
| RESPIRATORY PATHOGENS PANEL BY PCR | PARAINFLUENZA 3 PCR | - | 105489 |
| RESPIRATORY PATHOGENS PANEL BY PCR | PARAINFLUENZA 4 PCR | - | 105489 |
| RESPIRATORY PATHOGENS PANEL BY PCR | RHINOVIRUS ENTEROVIRUS PCR | - | 105489 |
| RESPIRATORY PATHOGENS PANEL BY PCR | RSV PCR | - | 105489 |
| RESPIRATORY PATHOGENS PANEL BY PCR | SEASONAL CORONAVIRUS 229E PCR | - | 105489 |
| RESPIRATORY PATHOGENS PANEL BY PCR | SEASONAL CORONAVIRUS HKU1 PCR | - | 105489 |
| RESPIRATORY PATHOGENS PANEL BY PCR | SEASONAL CORONAVIRUS NL63 PCR | - | 105489 |
| RESPIRATORY PATHOGENS PANEL BY PCR | SEASONAL CORONAVIRUS OC43 PCR | - | 105489 |
| SARS COV 2, PCR | SARS COV 2 PCR | - | 422027 |
| SARS COV-2 PCR + INFLUENZA A/B AND RSV BY PCR | INFLUENZA A PCR | - | 429318 |
| SARS COV-2 PCR + INFLUENZA A/B AND RSV BY PCR | INFLUENZA B PCR | - | 429318 |
| SARS COV-2 PCR + INFLUENZA A/B AND RSV BY PCR | RSV PCR | - | 429318 |
| SARS COV-2 PCR + INFLUENZA A/B AND RSV BY PCR | SARS COV 2 PCR | - | 429318 |
| SARS COV-2 PLUS FLU A & B PANEL (POCT) LIAT | INFLUENZA A PCR (POCT) LIAT | - | 427142 |
| SARS COV-2 PLUS FLU A & B PANEL (POCT) LIAT | INFLUENZA B PCR (POCT) LIAT | - | 427142 |
| SARS COV-2 PLUS FLU A & B PANEL (POCT) LIAT | SARS COV-2 PCR (POCT) LIAT | - | 427142 |
| SARS-COV-2 RAPID PCR | SARS COV 2 PCR | - | 453364 |

**Figure S1:** Temporal trends for emergency department asthma and influenza-like-illness visits, and selected environmental exposures, July 2018-July 2023


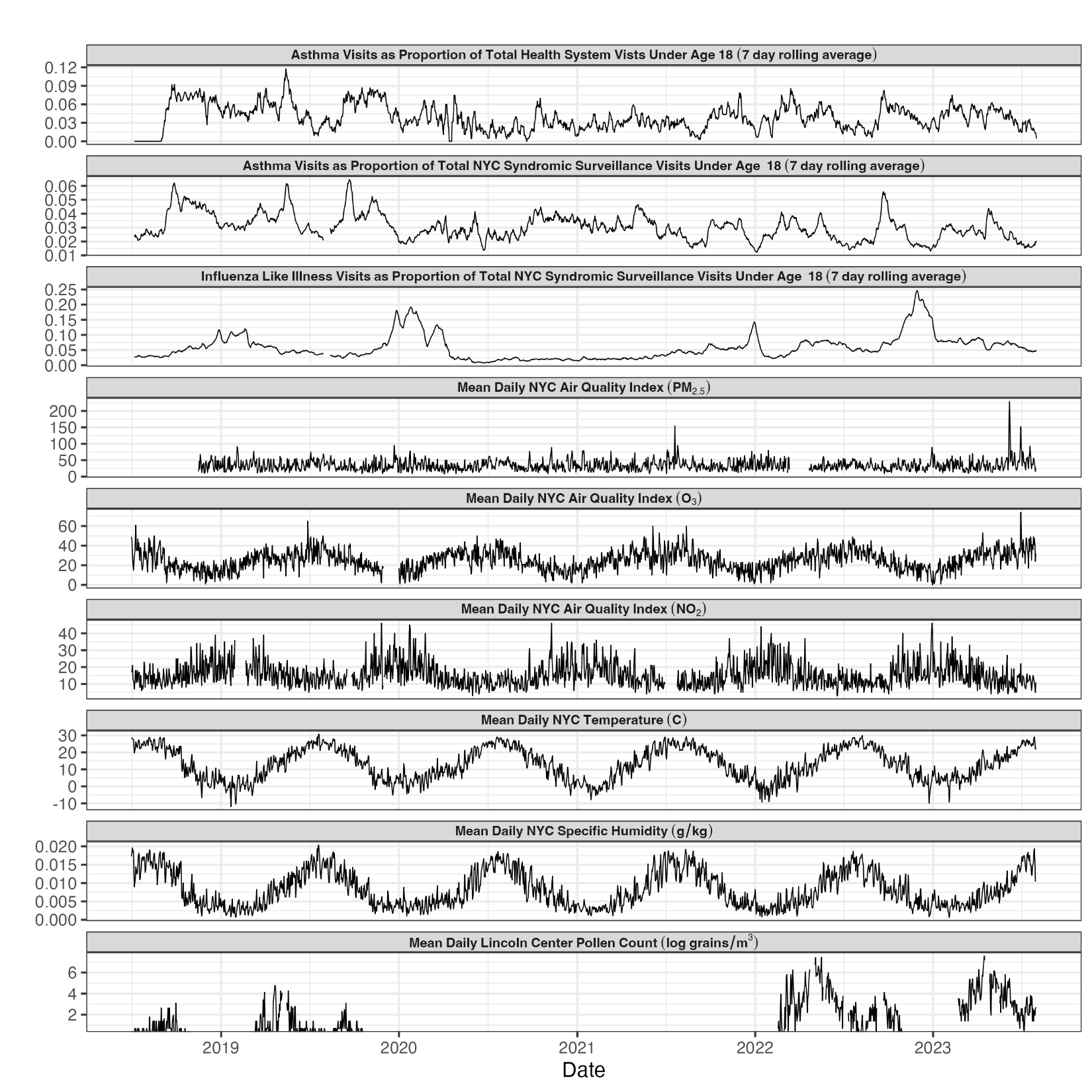


Note: Pollen count data were not collected during 2020-2021 due to the COVID-19 pandemic.

| Table S2. Demographics of children ages 5-17 years requiring intensive care unit (ICU) admission, who received viral testing, in a large New York City health system, school years from 2018-2023 | | | | | | | | | |
| --- | --- | --- | --- | --- | --- | --- | --- | --- | --- |
|  | **Period 1** | **Period 2** | | | **Period 3** | **Period 4** | | **Period 5** |  |
| School year (dates) | 9/5/18-6/26/19 | 9/5/19-6/25/20 | | | 9/16/20-6/25/21 | 9/13/21-6/27/22 | | 9/8/22-6/27/23 |  |
|  |  | |  |  | | |  |  |  |
| **Patient Information** | ***N*** | | | | | | | | ***p*** |
| Total Unique Patients | 54 | | 42 | 82 | | | 108 | 93 | <0.0001 |
| **Sex** | ***N (%)*** | | | | | | | |  |
| Male | 31 (57.4%) | | 18 (42.9%) | 41 (50%) | | | 63 (58.3%) | 47 (50.5%) | 0.420 |
| Female | 23 (42.6%) | | 24 (57.1%) | 41 (50%) | | | 45 (41.7%) | 46 (49.5%) | 0.420 |
| **Race-Ethnicity** | ***N (%)*** | | | | | | | |  |
| Black | 12 (22.2%) | | 13 (31%) | 30 (36.6%) | | | 28 (25.9%) | 34 (36.6%) | 0.443 |
| White | 11 (20.4%) | | 4 (9.5%) | 6 (7.3%) | | | 15 (13.9%) | 8 (8.6%) |  |
| Asian-American and Pacific Islander | 3 (5.6%) | | 5 (11.9%) | 8 (9.8%) | | | 5 (4.6%) | 4 (4.3%) |  |
| Other | 5 (9.3%) | | 6 (14.3%) | 8 (9.8%) | | | 13 (12.0%) | 14 (15.1%) |  |
| Hispanic/Latino | 23 (42.6%) | | 14 (33.3%) | 29 (35.4%) | | | 46 (42.6%) | 31 (33.3%) |  |
| Unknown | 0 (0%) | | 0 (0%) | 1 (1.2%) | | | 1 (0.9%) | 2 (2.2%) |  |
| **Insurance** | ***N (%)*** | | | | | | | |  |
| Medicare | 0 (0%) | | 0 (0%) | 1 (1.2%) | | | 1 (0.9%) | 1 (1.1%) | 0.547 |
| Medicaid | 41 (75.9%) | | 32 (76.2%) | 53 (64.6%) | | | 80 (74.1%) | 68 (73.1%) |  |
| Commercial | 32 (59.3%) | | 17 (40.5%) | 44 (53.7%) | | | 57 (52.8%) | 50 (53.8%) |  |
| Self Pay | 0 (0%) | | 1 (2.4%) | 0 (0%) | | | 0 (0%) | 0 (0%) |  |
| Other | 0 (0%) | | 1 (2.4%) | 0 (0%) | | | 3 (2.8%) | 2 (2.2%) |  |

Note: Statistical tests are χ^2^, except where expected values are <5, in which case they are Fisher’s exact test.

*Table S3: Number of viral tests, by type of test, administered in pediatric (ages 5-17 years) asthma emergency department and hospital encounters in a large New York City health system, by school year, 2018-2023.*

|  | **Period 1** | **Period 2** | **Period 3** | **Period 4** | **Period 5** |
| --- | --- | --- | --- | --- | --- |
| School year (dates) | 9/5/18-6/26/19 | 9/5/19-6/25/20 | 9/16/20-6/25/21 | 9/13/21-6/27/22 | 9/8/22-6/27/23 |
| **Test** | ***N*** | | | | |

| Multiplex PCR* | 1 | 17 | 10 | 88 | 77 |
| --- | --- | --- | --- | --- | --- |
| **3 or 4-plex PCR** |  |  |  |  |  |
| Influenza A | 130 | 114 | 146 | 338 | 576 |
| Influenza B | 130 | 115 | 146 | 341 | 577 |
| RSV | 130 | 115 | 146 | 341 | 579 |
| SARS-CoV-2 | N/A | 8 | 179 | 461 | 617 |
| **LIAT** |  |  |  |  |  |
| Influenza/RSV | 4 | 6 | 18 | 63 | 67 |
| SARS-CoV-2 | N/A | N/A | 12 | 63 | 67 |

Note: PCR: Polymerase chain reaction. Multiplex PCR includes the following viral targets: Adenovirus, Bordetella pertussis, Chlamydophila pneumoniae, Metapneumovirus, Mycoplasma pneumoniae, Parainfluenzas 1-4, Rhinovirus enterovirus, Influenza A, Influenza B, Respiratory syncytial virus, and after Period 2, SARS-CoV-2. In periods 4 and 5, it also includes Seasonal coronavirus 229e, Seasonal coronavirus HKU1, Seasonal coronavirus Nl63, and Seasonal coronavirus OC43.
